# Retraction Note: Activation of c-Src tyrosine kinase mediated the degradation of occludin in ventilator-induced lung injury

**DOI:** 10.1186/s12931-024-02692-7

**Published:** 2024-01-24

**Authors:** Tao Zhao, Mengjie Liu, Changping Gu, Xin Wang, Yuelan Wang

**Affiliations:** 1 Department of Anesthesiology, Qianfoshan Hospital, Shandong University, No. 16766 Jingshi Road, Jinan, 250014 Shandong Province China; 2 Department of Anesthesiology, Jinan Fifth People’s Hospital, Ji’nan, Shandong China

The Editors-in-Chief retracted this article because of concerns regarding a number of figures presented in this work. These concerns call into question the integrity of the data and the article’s overall scientific soundness. An investigation conducted after its publication discovered instances of image overlap both between images in this work and with images in [1].

The Editors-in-Chief therefore no longer have confidence in the integrity of the research presented in this article.

The authors disagree to the retraction.
